# Comparative study of two minimally invasive transforaminal interbody fusions for single-segment lumbar disc herniation: a retrospective study

**DOI:** 10.3389/fsurg.2026.1818487

**Published:** 2026-05-29

**Authors:** Yanhao Ge, Shixiang Wu, Xuequan Zhao, Jian Zhang, Gang Hu, Yafeng Zhang

**Affiliations:** 1 Nanjing University of Chinese Medicine, Nanjing, China; 2 Department of Orthopedics, Wuxi TCM Hospital Affiliated to Nanjing University of Chinese Medicine, Wuxi, China

**Keywords:** clinical efficacy, HBL, imaging indicators, LDH, MIS-TLIF, UBE-TLIF

## Abstract

**Objective:**

In this study, we aimed to evaluate and compare the clinical efficacy of unilateral biportal endoscopic transforaminal lumbar interbody fusion (UBE-TLIF) and minimally invasive transforaminal lumbar interbody fusion (MIS-TLIF) for the treatment of single-level lumbar disc herniation (LDH).

**Methods:**

A total of 60 patients who underwent surgery between January 2023 and January 2024 were retrospectively enrolled and divided into two groups based on the surgical technique: UBE-TLIF (*n* = 34) and MIS-TLIF (*n* = 26). Efficacy was evaluated using perioperative indicators, follow-up results, and imaging results.

**Results:**

Following surgery, both groups demonstrated significant longitudinal improvements in visual analog scale (VAS), Oswestry Disability Index (ODI), and Japanese Orthopaedic Association (JOA) scores compared with preoperative levels (*P* < 0.05). However, no statistically significant intergroup differences were observed at 1 week, 3 months, and the final follow-up (*P* > 0.05). Although the duration of hospital stay was comparable between the two groups, the operative time was significantly longer in the UBE-TLIF group (*P* < 0.05). Notably, the UBE-TLIF group had significantly lower values for hidden blood loss (HBL), visible blood loss (VBL), and total blood loss (TBL) than the MIS group (*P* < 0.05). Regarding radiographic parameters, both groups showed significant intragroup improvements in intervertebral space height, Cobb angle, and lumbar lordosis (LL) at 1 month and the final follow-up compared with preoperative levels (*P* < 0.05). Although the Cobb angle and LL showed statistical differences between the two groups at the final follow-up (*P* < 0.05), the absolute differences were minimal, indicating that both techniques achieved clinically comparable outcomes in sagittal alignment reconstruction.

**Discussion:**

Although UBE-TLIF requires a longer operative time than MIS-TLIF, it significantly reduces HBL, VBL, and TBL. Both surgical techniques yield favorable and comparable outcomes in terms of early pain relief, long-term clinical efficacy, and interbody fusion. Therefore, for middle-aged and elderly patients with single-level LDH, although both procedures are highly effective, UBE-TLIF demonstrates specific perioperative advantages in terms of blood conservation.

## 1 Introduction

The prevalence of lumbar disc herniation (LDH) has progressively affected younger populations because of shifts in modern occupational behaviors. LDH is a major cause of lumbosacral pain and significantly impairs daily activities, such as ambulation, thereby posing a substantial socioeconomic burden. Currently, vertebral fusion surgery is widely considered an effective therapeutic intervention that markedly improves patient symptoms and enhances quality of life (1).

Traditional open posterior lumbar fusion is a common surgical procedure. Posterior spinal column structures, including the interspinous ligaments, facet joints, spinous process, and joint capsules, are critically important for maintaining spinal stability, and their structural integrity largely dictates postoperative outcomes (2). However, conventional open surgery often causes extensive damage to the surrounding skin and paraspinal musculature, leading to iatrogenic posterior column instability. This instability can subsequently contribute to a cascade of complications including accelerated disc degeneration and facet joint pathology (1).

In recent years, minimally invasive lumbar interbody fusion techniques have garnered increasing attention as alternatives to traditional open posterior lumbar fusion (3). Transforaminal lumbar interbody fusion was first described by Harms and Rolinger in 1982 (4). Minimally invasive transforaminal lumbar interbody fusion (MIS-TLIF) is a viable treatment option for degenerative spinal conditions. Compared to traditional open surgery, MIS procedures are associated with smaller incisions, less intraoperative blood loss, and shorter postoperative recovery times (5). They are used for various lumbar degenerative diseases requiring fusion, such as degenerative LDH, lumbar spinal stenosis, spondylolisthesis, and instability (6).

Concurrently, with the application of arthroscopic technology in spine surgery, unilateral biportal endoscopic transforaminal lumbar interbody fusion (UBE-TLIF) has become popular for the management of LDH (7). The UBE-TLIF technique utilizes two distinct portals on one side, one for visualization and the other for working instruments, which facilitates enhanced maneuverability for the surgeon. Although simple decompression or microdiscectomy remains the gold standard treatment for uncomplicated LDH, transforaminal lumbar interbody fusion (TLIF) is strictly indicated in a highly specific subset of patients. In clinical practice, fusion is reserved for patients with LDH exhibiting concurrent segmental instability; severe foraminal stenosis requiring extensive facet joint resection (>50%), which inevitably leads to iatrogenic instability; or recurrent disc herniations accompanied by predominant mechanical low back pain (8). In these complex cases, MIS-TLIF and UBE-TLIF provide adequate neural decompression and immediate stabilization.

In this study, we aimed to compare the perioperative parameters, hidden blood loss (HBL), and postoperative pain scores [visual analog scale (VAS), Oswestry Disability Index (ODI), and Japanese Orthopaedic Association (JOA)] between MIS-TLIF and UBE-TLIF. By evaluating the clinical efficacy and postoperative recovery status of MIS-TLIF and UBE-TLIF in treating single-level LDH in middle-aged and older patients, we sought to provide a valuable reference for selecting an optimal surgical strategy.

## 2 Materials and methods

### 2.1 Inclusion and exclusion criteria

The inclusion criteria were as follows: patients with (1) a clear preoperative diagnosis of single-level LDH, (2) no significant relief after a minimum of 6 months of conservative rehabilitation treatment, and (3) LDH accompanied by explicit indications for interbody fusion. Specifically, these indications included: documented segmental instability; severe foraminal or lateral recess stenosis where adequate neural decompression would inevitably require substantial facet joint resection, thereby compromising postoperative stability; or recurrent disc herniations presenting with predominant mechanical back pain.

The exclusion criteria included: patients with (1) a history of lumbar surgery; (2) multilevel disc herniation; (3) concurrent lumbar tuberculosis, infection, or tumors; (4) massive herniation segments accompanied by symptoms of cauda equina syndrome; and (5) anticoagulant or antiplatelet medication received within 6 months prior to surgery.

### 2.2 General information

This study retrospectively analyzed the clinical data of patients diagnosed with LDH who were admitted to the Department of Spine Surgery between January 2023 and January 2024. A total of 60 patients who met the inclusion criteria were enrolled. According to the surgical technique used, the patients were divided into the UBE-TLIF (*n* = 34) and the MIS-TLIF (*n* = 26) groups. The study protocol was approved by the Ethics Committee, and informed consent was obtained from all patients.

### 2.3 Surgical technique

#### 2.3.1 UBE-TLIF group

1. Patient Positioning and Preparation: Following successful anesthesia, the patient was placed in the prone position. The surgical site was prepared with routine sterilization and draping.
2. Portal Establishment: The pedicles were first marked under C-arm fluoroscopy using a double-sided arched projection. An arch-guided puncture was performed approximately 1 cm from the bilateral vertebral body projection. After confirming the satisfactory puncture location of the arch under C-arm guidance, six guidewires were inserted, and their positions were re-confirmed fluoroscopically.
3. Incision and Initial Decompression: The surgical plane was determined. An oblique incision approximately 1 cm in length was made on the symptomatic side (ipsilateral to the predominant disc herniation) of the vertebral body projection, superior to the left pedicle. The endoscope was introduced via percutaneous subcutaneous soft tissue dissection. The facet joint and a portion of the lamina in the target intervertebral space were resected with endoscopic assistance.
4. Discectomy and Fusion: The nerve root was identified, and the nerve root canal was adequately decompressed (neurolysis). The nerve root was confirmed to be free from significant compression after decompression. The dura mater was retracted medially, and a radiofrequency electrode was used for hemostasis. Subsequently, the portals were exchanged using a cephalad incision as the viewing portal and a caudad incision as the working portal. The intervertebral space was prepared using alternating open reamers and spreaders using the UBE-TLIF technique under continuous endoscopic surveillance. The proximal lamina was trimmed to the inferior attachment of the ligamentum flavum on the proximal lamina. The superior border of the lower vertebral body lamina and the ligamentum flavum were also addressed. The entire ipsilateral ligamentum flavum layer was removed. An autologous bone graft was then implanted into the intervertebral space, followed by the insertion of the interbody cage. Satisfactory positioning of the cage was confirmed using fluoroscopy. Following interbody cage placement, bilateral percutaneous pedicle screws and connecting rods were percutaneously inserted and secured under C-arm fluoroscopic guidance to achieve rigid internal fixation. The surgical area was thoroughly irrigated with copious amounts of normal saline at the conclusion of the procedure.
5. Post-Procedure: The surgery was completed smoothly with satisfactory anesthesia. After dressing and bandaging the wound, the patient was transferred back to the ward.

#### 2.3.2 MIS-TLIF group

1. Patient Positioning and Localization: After general anesthesia, the patient was placed in the prone position. The intervertebral space of the pathological segment was localized using C-arm fluoroscopy, and the incision site was marked on the skin. After routine sterilization and sterile draping, a protective membrane strip was applied to the incision area.
2. Incision and Exposure: A longitudinal incision of approximately 3 cm in length was made paraspinally on the symptomatic side. A muscle-splitting approach was used to expose the ipsilateral facet joint and the base of the transverse processes of the affected segment, thereby minimizing paraspinal muscle stripping and trauma.
3. Decompression and Interbody Preparation: The inferior articular process of the upper vertebra and the superior articular process of the lower vertebra were resected using an osteotome, followed by removal of the ligamentum flavum. While meticulously protecting the nerve roots and dural sac, the annulus fibrosus was incised, the nucleus pulposus was removed, and the intervertebral space was thoroughly curetted to ensure clearance of residual annulus and nucleus pulposus. The adjacent vertebral endplate cartilage was scraped. Intervertebral spreaders were sequentially used to distract and mobilize the intervertebral space. An appropriately sized interbody fusion cage (containing autologous decompression bone) was placed at the anterior aspect of the intervertebral space. Subsequently, bilateral pedicle screws were placed percutaneously under C-arm fluoroscopic guidance, confirming optimal placement of the pedicle screws (internal fixation) and interbody fusion cage.
4. Closure: The incision was irrigated repeatedly. The lumbodorsal fascia, subcutaneous tissue, and skin were sequentially closed. After dressing application, the patient was transferred back to the ward.

#### 2.3.3 Postoperative management and efficacy evaluation criteria

Postoperatively, all patients in both groups were maintained on strict bed rest, and excessive activity was avoided. Patients received close monitoring and nursing care, including continuous monitoring of vital signs, inspection of the surgical incision and drainage tubes, and assessment of neurological function and pain levels. Symptomatic and supportive treatments such as pain management at the surgical site, infection prevention, and promotion of incision healing were administered based on the individual patient's condition. Patients were required to wear a lumbar brace while ambulating.

The following parameters were recorded and compared between the two groups: operative time, intraoperative blood loss, occult blood loss, postoperative drainage volume, postoperative hospital stay, and early complications. To evaluate clinical efficacy, low back pain and leg pain VAS scores, ODI, and JOA scores were assessed and compared preoperatively, 1 week postoperatively, 3 months postoperatively, and at the final follow-up (12 months postoperatively).

**Figure 1:**
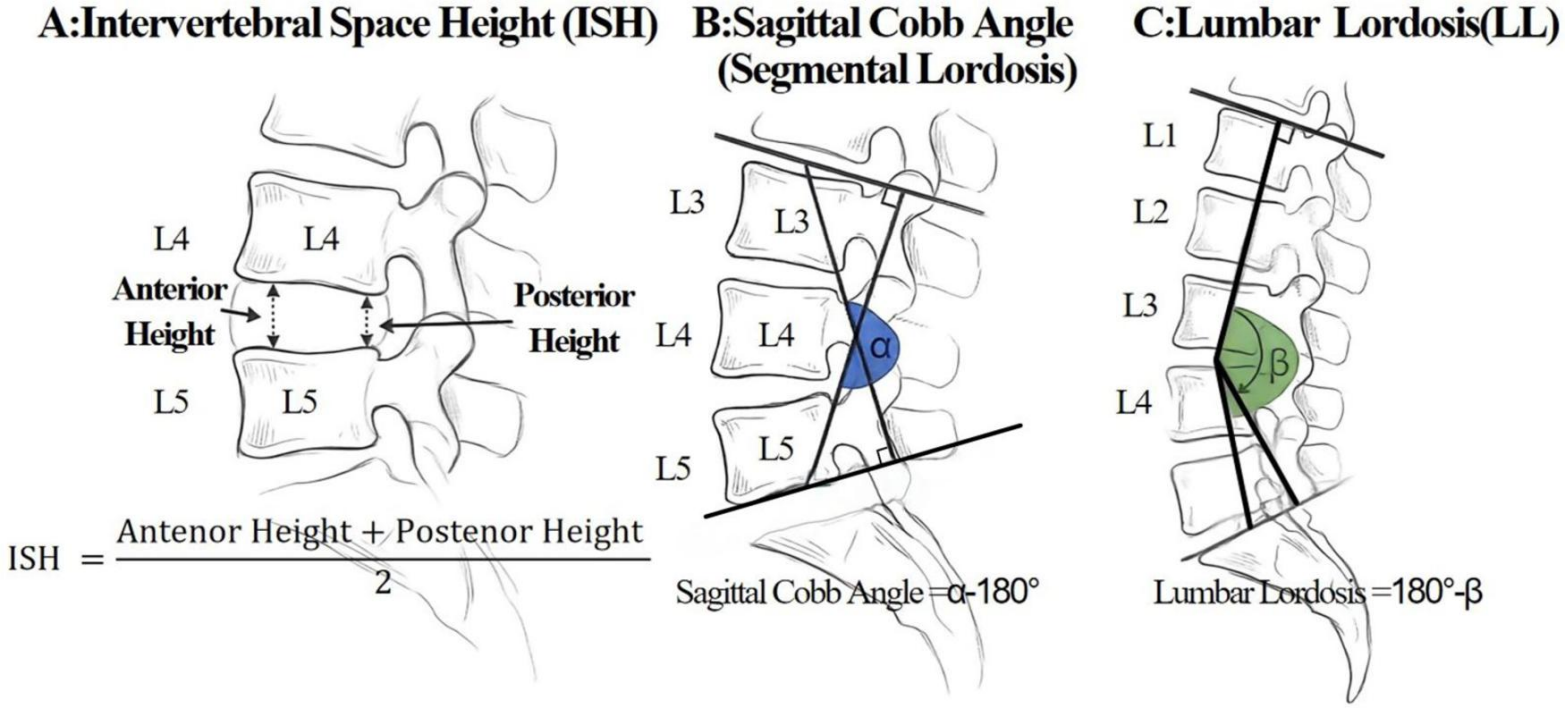
Schematic illustration of the radiographic measurement methods on lateral lumbar radiograph. **(A)** Intervertebral space height (ISH) is calculated as the average of the anterior and posterior disc heights, **(B)** sagittal Cobb angle represents the segmental lordosis at the surgical level, and **(C)** lumbar lordosis (LL) is measured between the superior endplate of L1 and the superior endplate of S1.

Standard anteroposterior and lateral lumbar spine radiographs were obtained preoperatively, 1 month postoperatively, and at the final follow-up (12 months postoperatively). To ensure measurement accuracy and mitigate assessment bias, all radiographic parameters were measured independently by two experienced spine surgeons who were not involved in the surgical procedures and blinded to the patient groups. The measurements were performed digitally using our hospital's Picture Archiving and Communication System (PACS). The radiographic parameters were strictly defined and measured as follows: (1) intervertebral space height (ISH), calculated as the mean value of the anterior and posterior heights of the operated intervertebral disc space; (2) sagittal Cobb angle (segmental lordosis), defined as the angle subtended by a line drawn parallel to the superior endplate of the upper adjacent vertebra and a line parallel to the inferior endplate of the lower adjacent vertebra at the fused segment; and (3) lumbar lordosis (LL), measured as the angle between the superior endplate of L1 and the superior endplate of S1. A detailed schematic illustrating the measurement methods for these parameters is provided in Figure 1.

#### 2.3.4 Statistical methods

Statistical analyses were performed using SPSS software (version 27.0). Measurement data are presented as the mean ± standard deviation. For normally distributed data, independent samples *t*-tests were used for comparisons between the two groups, and one-way analysis of variance was used for comparisons among different time points within a group. The Mann–Whitney *U* test was used for non-normally distributed data. Categorical data were compared using the chi-squared test or Fisher's exact test, as appropriate. Ordinal data were compared between the two groups using the Mann–Whitney *U* test, whereas comparisons within groups across multiple time points were performed using the Friedman test for related samples. Statistical significance was set at *P* < 0.05.

## 3 Results

All patients in both groups successfully underwent surgery and were followed up; all surgical incisions achieved Grade A healing.

### 3.1 General data

**Figure 2:**
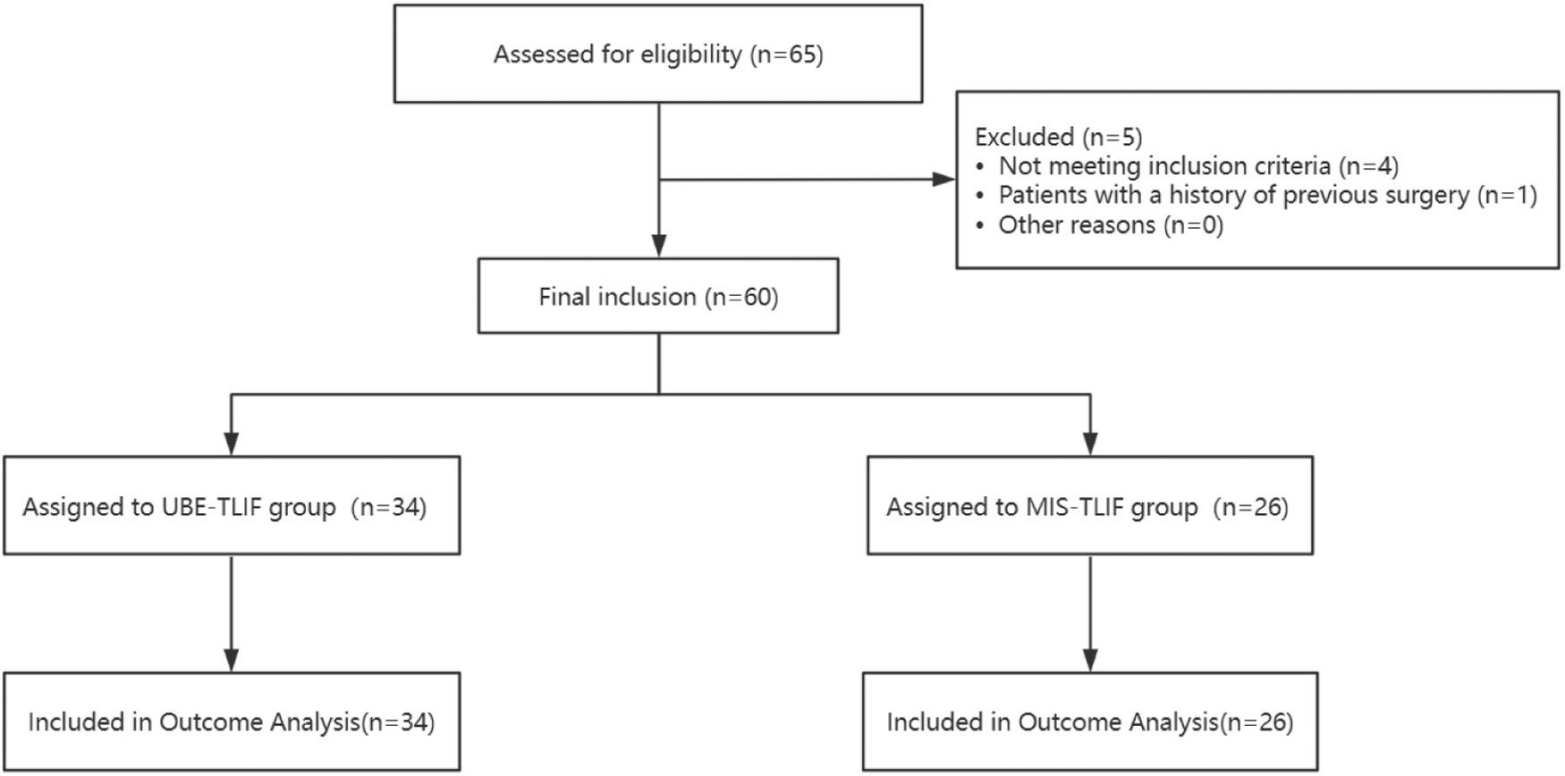
Flow chart of test grouping.

We initially screened 65 patients with LDH. After applying the predefined inclusion and exclusion criteria, five patients were excluded, including four not meeting the inclusion criteria and one with a history of surgery. Finally, 60 eligible patients were included and allocated to two surgical groups: UBE-TLIF (*n* = 34) and MIS-TLIF (*n* = 26). All enrolled patients completed the 12-month follow-up and were included in the outcome analysis without any loss to follow-up (Figure 2). The baseline characteristics of the two groups, including age, sex, BMI, surgical segment, smoking status, diabetes mellitus, and American Society of Anesthesiologists classification, were compared. No statistically significant differences were found between the UBE group and MIS group (*P* > 0.05) (Table 1).

**Table 1:** Comparison of general data between the two groups before treatment.

Parameter	UBE-TLIF (*n* = 34)	MIS-TLIF (*n* = 26)	*P*-value
Age (years, *x̅* ± *s*)	51.3 ± 10.6	53.6 ± 11.3	0.393
Sex (male/female, *n*)	22/12	17/9	0.151
BMI (kg/m^2^, *x̅* ± *s*)	24.20 ± 3.14	24.86 ± 3.61	0.424
Surgical segment (*n*) (L3–4/L4–5/L5–S1)	6/13/15	3/9/14	0.568
Smoking status (yes/no, *n*)	15/19	11/15	0.888
Diabetes mellitus (yes/no, *n*)	10/24	7/19	0.832
ASA classification (I/II/III, *n*)	6/28/0	2/24/0	0.298

UBE-TLIF, unilateral biportal endoscopic transforaminal lumbar interbody fusion; MIS-TLIF, minimally invasive transforaminal lumbar interbody fusion; BMI, body mass index; ASA, American Society of Anesthesiologists.

### 3.2 Perioperative data

HBL was calculated as total blood loss (TBL) minus visible blood loss (VBL) plus transfusion volume. Therefore, calculating HBL required the determination of TBL and VBL. TBL was calculated using the formula described by Gross et al.: $TBL\;(mL)\;=\;Patient^{\prime }s\;Blood\;\;Volume\;(in\;L)\;\times \;(Preoperative$ Hct−PostoperativeHct)/[(PreoperativeHct+Postoperative Hct)/2]×1000 (9). In accordance with the guidelines, hematocrit values were measured preoperatively and on postoperative day 1, after hemodynamic stabilization (9). PBV was estimated based on the patient's sex, height, and weight using the Nadler et al. formula: $PBV\;(L)\;=\;k1\;\times \;height\;(m{)}^{3}\;+\;k2\;\times \;weight\;(kg)\;+\;k3$. The constants were *k*1 = 0.3669, *k*2 = 0.03219, and *k*3 = 0.6041 for males, and *k*1 = 0.3561, *k*2 = 0.03308, and *k*3 = 0.1833 for females (10).

Because all patients had a postoperative drainage tube placed, VBL was approximated as the intraoperative blood loss plus postoperative drainage volume. Intraoperative blood loss comprised the volume in the suction bottle (minus the irrigation fluid used) and the blood absorbed by gauzes and sponges (minus the weight of the dry gauzes and sponges used). Since no blood transfusions were administered during or after surgery, HBL was ultimately calculated as follows: HBL=TBL−IntraoperativeBloodLoss−DrainageVolume. Consequently, obtaining the HBL value required calculating TBL from the change in hematocrit and determining VBL from the intraoperative blood loss and drainage volume, with the difference between TBL and VBL attributed to HBL.

The operative time was slightly longer in the UBE-TLIF group than in the MIS-TLIF group. However, the time to ambulation and the postoperative hospital stay were slightly shorter in the UBE-TLIF group than in the MIS-TLIF group. These differences were not statistically significant. The HBL was significantly lower in the UBE-TLIF group (714.74 ± 46.444 mL) than in the MIS-TLIF group (948.27 ± 75.828 mL). Furthermore, both TBL and VBL were significantly lower in the UBE-TLIF group than in the MIS-TLIF group. A statistically significant difference was also observed in the percentage of HBL relative to TBL between the two groups. No postoperative complications were observed in either group (Table 2).

**Table 2:** Comparison of perioperative parameters between the two groups.

Parameter	UBE-TLIF (*n* = 34)	MIS-TLIF (*n* = 26)	*P*-value
Operative time (min, *x̅* ± *s*)	156.5 ± 16.6	135.6 ± 15.7	*P* < 0.001
Total blood loss (TBL, mL)	798.47 ± 43.102	1,105.38 ± 88.010	*P* < 0.001
Visible blood loss (VBL, mL)	82.25 ± 12.410	157.12 ± 20.984	*P* < 0.001
Hidden blood loss (HBL, mL)	714.74 ± 46.444	948.27 ± 75.828	*P* < 0.001
HBL/TBL ratio (%)	89.514	85.787	*P* < 0.001
Time to ambulation (days, *x̅* ± *s*)	3.4 ± 1.2	4.1 ± 1.6	0.358
Hospital stay (days, *x̅* ± *s*)	8.4 ± 1.7	8.9 ± 2.3	0.163

UBE-TLIF, unilateral biportal endoscopic transforaminal lumbar interbody fusion; MIS-TLIF, minimally invasive transforaminal lumbar interbody fusion.

### 3.3 Comparison of VAS, ODI and JOA before and after surgery

Preoperatively, there were no statistically significant differences between the UBE-TLIF group and the MIS-TLIF group regarding VAS scores for back and leg pain (*P* > 0.05). Postoperative VAS scores for both low back pain and leg pain significantly decreased compared with preoperative levels in both groups, and these differences were statistically significant (*P* < 0.05). Regarding intergroup comparisons, there were no statistically significant differences in VAS scores at 1 week, 3 months, and the final follow-up (*P* > 0.05).

Regarding the ODI, no significant intergroup differences were noted preoperatively (*P* > 0.05). Postoperative ODI scores showed significant improvement compared with preoperative scores in both groups (*P* < 0.05). No statistically significant differences in ODI were observed between the two groups at the 3-month and final follow-ups (*P* > 0.05).

Similarly, preoperative JOA scores were comparable between the two groups. Postoperative JOA scores increased significantly from preoperative levels in both groups, with statistically significant differences (*P* < 0.05). Intergroup comparisons of the JOA scores at the 3-month and final follow-ups revealed no statistically significant differences (*P* > 0.05) (Table 3).

**Table 3:** Comparison of VAS, ODI, and JOA scores before and after surgery between the two groups (*x̅* ± *s*).

Parameter	UBE-TLIF (*n* = 34)	MIS-TLIF (*n* = 26)	*P*-value
Leg pain VAS (points)			
Preoperatively	6.97 ± 0.797	6.88 ± 0.864	0.691
1 week postoperatively	3.53 ± 1.022	3.62 ± 0.941	0.728
3 months postoperatively	2.47 ± 0.615	2.50 ± 0.648	0.858
Final follow-up	1.38 ± 0.493	1.42 ± 0.504	0.755
Low back pain VAS (points)			
Preoperatively	5.12 ± 0.880	4.96 ± 0.821	0.945
1 week postoperatively	4.15 ± 0.702	4.12 ± 0.588	0.801
3 months postoperatively	2.50 ± 0.508	2.46 ± 0.508	0.772
Final follow-up	1.53 ± 0.507	1.54 ± 0.508	0.946
ODI (%)			
Preoperatively	32.21 ± 3.867	31.31 ± 3.586	0.362
3 months postoperatively	24.53 ± 2.339	24.15 ± 2.185	0.529
Final follow-up	22.38 ± 1.181	22.42 ± 1.102	0.892
JOA (points)			
Preoperatively	14.79 ± 2.071	15.15 ± 1.870	0.490
3 months postoperatively	20.85 ± 2.190	20.54 ± 2.249	0.588
Final follow-up	21.06 ± 1.496	20.81 ± 1.415	0.512

UBE-TLIF, unilateral biportal endoscopic transforaminal lumbar interbody fusion; MIS-TLIF, minimally invasive transforaminal lumbar interbody fusion; VAS, visual analog scale; ODI, Oswestry Disability Index; JOA, Japanese Orthopaedic Association.

### 3.4 Radiological outcomes

#### 3.4.1 Intragroup comparison

In both the UBE-TLIF and MIS-TLIF groups, the ISH, sagittal Cobb angle, and LL showed statistically significant improvements between the preoperative assessment and measurements taken 1 month post-operation and at the final follow-up (*P* < 0.05).

#### 3.4.2 Intergroup comparison

No statistically significant differences were observed between the UBE-TLIF and MIS-TLIF groups for the ISH, sagittal Cobb angle, or LL in the preoperative assessments or at the 1-month postoperative follow-up. A statistically significant difference was detected between the two groups in both the sagittal Cobb angle and the LL at the final follow-up (*P* < 0.05). However, the absolute numerical differences were exceptionally small (<1 degree) (Table 4).

**Table 4:** Comparison of auxiliary examination parameters between the two groups (*x̅* ± *s*).

Parameter	UBE-TLIF (*n* = 34)	MIS-TLIF (*n* = 26)	*P*-value
ISH (mm)			
Preoperatively	8.24 ± 0.78	8.28 ± 0.84	0.840
1 month postoperatively	11.24 ± 0.81	11.47 ± 0.77	0.283
Final follow-up	10.60 ± 0.67	10.75 ± 0.68	0.386
Cobb angle (°)			
Preoperatively	8.50 ± 0.43	8.47 ± 0.47	0.799
1 month postoperatively	9.59 ± 0.28	9.58 ± 0.34	0.897
Final follow-up	9.32 ± 0.17	9.22 ± 0.18	*P* < 0.05
LL (°)			
Preoperatively	46.61 ± 0.85	46.59 ± 0.92	0.923
1 month postoperatively	49.65 ± 0.86	49.82 ± 0.88	0.459
Final follow-up	50.31 ± 0.59	50.64 ± 0.64	*P* < 0.05

UBE-TLIF, unilateral biportal endoscopic transforaminal lumbar interbody fusion; MIS-TLIF, minimally invasive transforaminal lumbar interbody fusion; ISH, intervertebral space height.

#### 3.4.3 Fusion rate

The interbody fusion status was assessed by observing the fusion between the interbody cage and adjacent vertebral endplates on radiographs. The fusion rates were 91.3% (34 patients) and 89.6% (26 patients) in the UBE-TLIF and MIS-TLIF groups, respectively. Both surgical techniques demonstrated favorable fusion rates, and the difference in the fusion rates between the two groups was not statistically significant (*P* > 0.05).

**Figure 3:**
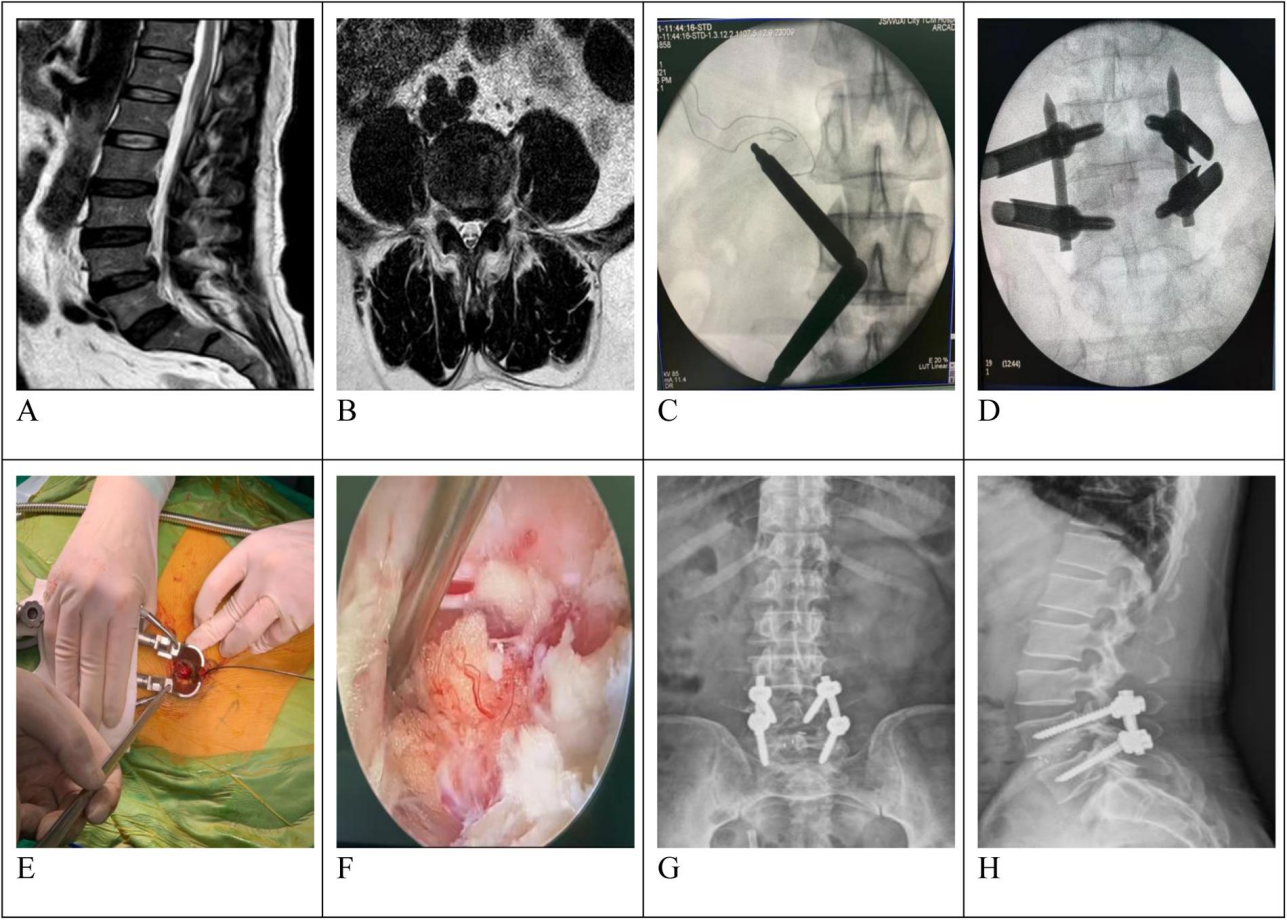
Illustrative case of a 52-year-old male patient with lumbar pathology at the L4-L5 level. **(A)** Preoperative sagittal T2-weighted magnetic resonance imaging (MRI) of the lumbar spine. **(B)** Preoperative axial MRI at the surgical segment. **(C)** Intraoperative fluoroscopic image for target segment localization. **(D)** Intraoperative photograph following the placement of the pedicle screw-rod construct. **(E)** Intraoperative macroscopic view of the surgical field. **(F)** Intraoperative endoscopic view demonstrating the dissection and decompression of the nerve root. **(G)** Postoperative anteroposterior radiograph. **(H)** Postoperative lateral radiograph.

Radiographic examples are shown in Figure 3.

## 4 Discussion

Posterior lumbar interbody fusion is an effective surgical technique for treating various lumbar spine pathologies such as LDH, spondylolisthesis, and lumbar spinal stenosis (11). While open procedures, including traditional posterior lumbar interbody fusion, remain viable, many patients, especially elderly patients with declining physical function, may not tolerate them well because of associated complications. Consequently, advanced techniques are needed for minimally invasive spine surgery. Both MIS-TLIF and UBE-TLIF are viable options for treating degenerative spinal conditions. Compared to open surgery, these minimally invasive approaches are associated with less tissue trauma, reduced intraoperative blood loss, and shorter postoperative recovery times.

Recently, numerous clinical studies and meta-analyses have confirmed the efficacy and safety of minimally invasive lumbar interbody fusion (12). Some studies have indicated that adjacent segment instability can occur following lumbar fusion because of alterations in load-bearing dynamics. Vertebral instability is a pathological condition characterized by failure of the spinal motion segment to maintain normal positional relationships under physiological loads (13). MIS-TLIF, which typically employs a bilateral approach, is effective in decompression and reduction. This bilateral access allows effective bilateral decompression of the isthmus and lateral recess and facilitates further manipulation and release of the intervertebral space, effectively aiding in the removal of herniated nucleus pulposus tissue to achieve adequate decompression with minimal residual fragments. In contrast, UBE-TLIF offers enhanced visualization of neural elements, degenerative surrounding structures, and the often-congested epidural venous plexus. This superior visualization is crucial for achieving optimal surgical outcomes.

Since 2000, spinal surgeons have become increasingly concerned about HBL. Lei et al. reported an average HBL of 449 ± 191 mL in posterior lumbar interbody fusion, accounting for 44.2 ± 16.6% of the TBL (14). With the growing popularity of minimally invasive spine surgery, researchers have gradually recognized the role of HBL. Wu et al. reported an average HBL of 282 ± 163 mL in percutaneous kyphoplasty (15), while Zhou et al. documented an average HBL of 488.4 ± 294.0 mL in MIS-TLIF, constituting 52.5% of the TBL (16). HBL has been demonstrated to increase TBL, exacerbate postoperative hemoglobin decline, and increase transfusion requirements. If not managed properly, it may lead to perioperative complications such as delayed wound healing, increased risk of infection, and prolonged recovery time (17). Our findings indicate that UBE-TLIF resulted in significantly lower intraoperative blood loss compared to MIS-TLIF, despite a longer operative time. This suggests that operative time is not the primary determinant of HBL, consistent with previous studies (18). From a technical perspective, Unlike MIS-TLIF, which requires a 3-cm incision and some degree of paraspinal muscle splitting within the tubular retractor, UBE-TLIF utilizes two independent, small portals, which further minimizes the disruption of the muscle attachment points and the dorsal rami of the spinal nerves (19). It can also be inferred that UBE-TLIF provides better soft tissue protection than MIS-TLIF (20), which is a plausible explanation for our HBL findings.

Regarding functional outcomes, The VAS, ODI, and JOA scores are well-established instruments for evaluating functional recovery and postoperative outcomes after lumbar spine surgery (21). Our data indicated that both groups showed significant improvements, with significant decreases in lower back and leg pain VAS and ODI scores and a significant increase in JOA scores at various postoperative time points. Both surgical techniques achieved significant and comparable pain relief throughout the follow-up period. However, given the aging population and the increasing number of elderly patients with compromised baseline vital signs (22), strict perioperative blood loss control is imperative. For these patients, UBE-TLIF is recommended to further reduce perioperative bleeding and adverse events.

Recent meta-analyses corroborate that UBE-TLIF significantly mitigates intraoperative blood loss compared with MIS-TLIF (23). Concurrently, our results demonstrated that UBE-TLIF significantly reduced TBL, VBL, and HBL compared with MIS-TLIF. This could be attributed to the unique technical characteristics of the biportal endoscopic system. Continuous irrigation with saline creates hydrostatic pressure that helps suppress venous bleeding from the epidural plexus (24, 25). Moreover, magnified, high-definition visualization allows the surgeon to identify and coagulate small bleeding points more precisely than is possible using a tubular retractor. For elderly patients and those with multiple comorbidities, this reduction in HBL is of paramount clinical importance in maintaining hemodynamic stability and promoting systemic recovery.

Regarding radiographic outcomes, although the absolute differences in the Cobb angle and LL were minimal, the ability of UBE-TLIF to achieve a comparable or slightly more refined restoration of sagittal alignment underscores its technical reliability. UBE-TLIF provides a broader and more dynamic viewing angle, facilitating more thorough discectomy and endplate preparation under direct vision. This “close-up” visualization ensures that the interbody cage is placed in the optimal biomechanical position, which is fundamental for long-term segmental stability and successful fusion.

Our study has several limitations. First, the sample size was relatively small. Studies with smaller sample sizes are more prone to overestimating the treatment effects than those with larger sample sizes. Second, this study primarily focused on short-term parameters to facilitate enhanced recovery after surgery. A longer follow-up period (e.g., 2 years) is necessary to evaluate the long-term clinical outcomes of the UBE-TLIF and MIS-TLIF groups. Third, all procedures were performed by a single, highly experienced surgeon. The outcomes may differ if surgeons with varying levels of experience are involved. Consequently, future investigations should include a larger sample size, long-term follow-up, and prospective randomized controlled trials to further validate the comparative merits of these two surgical techniques.

## 5 Conclusion

Based on our findings, both UBE-TLIF and MIS-TLIF, as minimally invasive techniques for single-level LDH, demonstrate significant clinical efficacy and safety; however, each has distinct advantages and disadvantages. Although UBE-TLIF is associated with a longer operative time than MIS-TLIF, it is superior in terms of time to return to normal work or daily activities, intraoperative blood loss, and postoperative drainage volume. Therefore, we believe that UBE-TLIF is a favorable surgical alternative, particularly in clinical scenarios in which perioperative blood conservation and minimal tissue trauma are of paramount importance.

## Data Availability

The original contributions presented in the study are included in the article/Supplementary Material, further inquiries can be directed to the corresponding authors.
